# Three Contrasting Accounts of Electronic Gambling Machine Related Harm: Impacts on Community Views Towards Gambling Policy and Responsibility

**DOI:** 10.1007/s10899-023-10206-1

**Published:** 2023-04-28

**Authors:** Dan Myles, Kerry O’Brien, Murat Yücel, Adrian Carter

**Affiliations:** 1 Turner Institute for Brain and Mental Health, School of Psychological Sciences, Faculty of Medicine Nursing and Health Sciences, Monash University, Melbourne, VIC Australia; 2 School of Social Sciences, Faculty of Arts, Monash University, Melbourne, VIC Australia

**Keywords:** Gambling, Harm minimization policy, Pre-commitment, Losses disguised as wins, Responsibility, Community attitudes

## Abstract

This study investigated whether there was community support for prominent gambling harm reduction policies, as well as perceived responsibility for electronic gambling machine (EGM) related harm in an Australian sample (n = 906). Using a randomised experimental design, we also explored whether these outcomes were influenced by three alternative explanations for EGM-related harm: a brain-based account of gambling addiction, an account that highlighted the intentional design of the gambling environment focused on the “losses disguised as wins” (LDWs), and a media release advocating against further government intervention in the gambling sector. We observed clear majority support for most policies presented, including mandatory pre-commitment, self-exclusion, and a $1 limit on EGM bets. A substantial majority of participants agreed that individuals, governments, and industry should be held responsible for EGM-related harm. Participants presented with the explanation of LDWs attributed greater responsibility for gambling-related harm to industry and government, less agreement that electronic gambling machines are fair, and more agreement that EGMs are likely to mislead or deceive consumers. There was some limited evidence of greater support for policy intervention in this group, including a blanket ban of EGMs, clinical treatment funded by gambling taxes, mass media campaigns, and mandatory pre-commitment for EGMs. We found no evidence that a brain-based account of gambling addiction substantially undermined support for policy intervention. We predicted that the information about LDWs and the brain-based account of EGM related harm would soften attributions of personal responsibility for gambling harm. Our results did not support either of these predictions.

**Supplementary Information:**

The online version contains supplementary material available at 10.1007/s10899-023-10206-1.

## Introduction

Mass media campaigns to inform the public about the risks associated with certain behaviours or products can play an important role in public health efforts to reduce harm (Hornik, 2002). In the case of tobacco, public awareness that smoking is associated with long term health consequences and that cigarettes are addictive was important in increasing smoking cessation (Brandt, 2007). Media campaigns that build awareness can also increase public support for public health policies, such as restrictions on where you could smoke and bans on cigarette advertising, that were critical in reducing smoking rates and changing social norms (Nathanson, 1999).

Campaigns to increase awareness of the harms associated with electronic gambling machines (EGMs) could also be effective in reducing gambling harm and galvanising public support for regulation or policy interventions that minimise EGM-related harm. The gambling environment, in particular the design of EGMs, contribute to gambling harm and addiction (Yücel et al., 2017). The gambling industry has played a major role in the creation of a gambling environment, including the design of highly reinforcing EGMs (Yücel et al., 2018), and other strategies that maintain or increase gambling and gambling harm (Schüll, 2012). Problem gambling awareness campaigns concerning the harms associated with EGMs may therefore include explanations of how EGMs have been designed to intensify reinforcement during gambling or to distort cognitions about gambling via structural characteristics, such as “losses disguised as wins” (LDWs), near miss events, or bonus features (Barton et al., 2017; Myles et al., 2018).

Campaigns may also seek to highlight the addictive potential of EGMs by appealing to neuroscientific research demonstrating the involvement of mesolimbic reward pathways and other brain regions related to fostering or maintaining substance addiction and harmful gambling behaviour (Fauth-Bühler et al., 2017; Murch & Clark, 2016). This line of research was also influential in the renaming and re-classification of gambling disorder (GD) under Substance-Related and Addictive Disorders in the DSM 5 (American Psychiatric Association, 2013; Hasin et al., 2013; Petry et al., 2014). Thus, a neurobiological account of GD is likely to increasingly inform the treatment and diagnosis of gambling disorder and be communicated to patients and their families.

This shift may align perspectives of GD with the influential framing of addiction as a chronic, relapsing brain disease, a position forcefully advocated for by the US National Institute of Drug Abuse (Leshner, 1997; Volkow et al., 2016). Advocates of this position have contended that it has been effective in challenging views that addiction occurs as a result of a moral failing or lack of will power (Dackis & O’Brien, 2005; Leshner, 1997; Volkow & Koob, 2015) and crucial in attaining important health policy changes, such as eligibility for medical insurance coverage for the costs of addiction treatment in the US (Volkow & Koob, 2015). However, these claims have been criticised for lacking empirical support (Hall et al., 2015) or the additional concern that neuroscientific accounts of gambling addiction may shift attributions of responsibility for gambling harm away from the gambling products or the gambling industry and towards individual gamblers or erode public support for policies aimed at curbing industry-led environmental determinates of gambling harm (Yücel et al., 2017). A narrow biomedical account of gambling addiction may also be used by industry groups to promote the perspective that current EGM arrangements are safe, by framing gambling harm as a mental health condition that only affects a minority of problem individuals, a strategy that has been used to successfully lobby governments to avoid policies targeted at regulating product design or industry practice (Livingstone & Woolley, 2007; Markham & Young, 2015; Panichi, 2013).

Many of the concerns and claims reviewed here have not been empirically tested within the context of gambling-related policy. To address this gap, we conducted an online survey with a randomised experimental design to explore the impact of three prominent accounts of EGM-related harm on the endorsement of public policy interventions intended to minimise gambling-related harm, and perceived responsibility for that harm. An explanation of these experimental conditions, and a set of exploratory hypotheses, related to this study aim is provided in the methods section below. We also sought to characterise the degree of overall community support, across all experimental conditions, for each of the harm reduction policies described in the survey, as well as perceived responsibility for EGM related harm.

## Methods

De-identified data, analysis scripts and all study materials, including all survey items and the image files and text displayed to each experimental group have been made available on the Open Science Framework (OSF).

### Procedure

Qualtrics were contracted to recruit a representative sample of individuals 18 years and older living in the Australian states of New South Wales and Victoria. Soft quotas were employed to ensure that sampling aligned with population statistics on age, gender, and location (metro/regional). Participants were invited to participate in the study via a web-link to our survey page hosted on the Qualtrics survey platform. Participants were randomised to one of four conditions: a non-intervention control condition, or one of three experimental conditions, described below.

Participants were instructed that the page would set a timer to ensure that they had “*enough time to read the article in full before continuing on”*. They were also told to expect questions about the content of the article later in the survey. To confirm that participants had satisfactorily attended to the intervention article, they were presented with an immediate comprehension check. This four-item multiple choice question instructed participants to select the response that best described the content of the article. The three incorrect multiple-choice responses were the same across the experimental conditions, while a fourth accurately summarised a crucial piece of prominent information from the article displayed.

Participants who answered correctly were directed to the main body of the survey. Those who answered incorrectly were presented with the following text: “*The option you selected was incorrect. Please be sure to read the whole article carefully so that you are able to answer the next question.”* These participants were again shown the intervention article page. Following this, they were asked to respond to another comprehension check with a new set of answers. All participants, including those who failed the second check, were directed to complete the main body of the survey and were included in all analyses. The purpose of these items was to encourage compliance and measure comprehension in each intervention. Exclusion of these participants would have introduced an attrition bias, as individuals in the control condition did not complete a comprehension check. Participants were then asked to respond to our survey items. All study procedures were approved by the Monash University Human Research Ethics Committee (MUHREC Project ID: 17815).

### Intervention

Participants in each experimental condition were instructed to read one of three short online articles written by the study authors. Each intervention was approximately the same length and displayed an image at approximately the same location, see supplementary materials for text and images used. No intervention was displayed to participants in the control condition. The “Brain” intervention was presented as an online news article, including a masthead from a widely read Australian newspaper. It emphasised the role of neurobiology in gambling disorder and addiction and included an image depicting brain imaging data as well as an interview with a fictious neuroscience researcher explaining how reward uncertainty, a defining feature of gambling, is thought to increase activity in the brain’s reward system. The “Design” intervention was presented in the same manner and contended that EGMs have been deliberately designed to provoke extended or repeated gambling. The article was based on existing news publications (Evershed et al., 2017; Livingstone, 2015) and included a number of quotes from interviews with “gambling industry insiders” taken directly from the book *Addiction by Design* (Schüll, 2012). The article also included a summary of research conducted by Dixon and colleagues (2010; also see Graydon et al., 2021, for a recent review) concerning a feature of EGM design they have described as “losses disguised as wins”, that lead consumers to misperceive certain losses as gains. Finally, the “Industry” intervention was presented as a media release presented on a fictious pro-gambling lobby group webpage. This intervention framed gambling-related harm as a relatively rare condition that is primarily a matter for individual responsibility and treatment, rather than government intervention. It argued that existing industry programs already minimise gambling harm, that industry provides substantial financial support for community programs and that any further policy intervention would infringe upon individual liberties and damage the economy.

### Measures

All survey items are described in more detail in the supplementary materials.

### Demographics

We collected demographic information on age, gender identity, state, local area type (e.g., major city, remote location), education level, employment status, and income, see Table 1.

**Table 1:** Sample demographic information

Demographic	Value	Count	Percent
State of residence	New South Wales	433	47.8
	Victoria	473	52.2
Locality	Major city	661	73.0
	Inner regional	144	15.9
	Outer regional	88	9.7
	Remote	12	1.3
	Very remote	1	0.1
Age group	18–24	77	8.5
	25–34	175	19.3
	35–44	165	18.2
	45–54	156	17.2
	55–64	145	16.0
	65 +	188	20.8
Gender	Female	468	51.7
	Male	436	48.1
	Non-binary	1	0.1
	Other – no text response	1	0.1
Education	Year 10 or below	83	9.2
	Year 11 or equivalent	33	3.6
	Year 12 or equivalent	133	14.7
	A trade, technical certificate or diploma	227	25.1
	Undergraduate university degree	268	29.6
	Postgraduate degree	162	17.9
Employment	Not employed, not looking for work	267	29.5
	Not employed—looking for work	96	10.6
	Currently stood down	11	1.2
	Casual	44	4.9
	Part time	149	16.4
	Full time	339	37.4
Income	Negative income	3	0.3
	Nil income	14	1.5
	$1–$99	8	0.9
	$100–$199	12	1.3
	$200–$299	15	1.7
	$300–$399	30	3.3
	$400–$599	95	10.5
	$600–$799	91	10.0
	$800–$999	77	8.5
	$1,000–$1,249	110	12.1
	$1,250–$1,499	66	7.3
	$1,500–$1,999	93	10.3
	$2,000–$2,499	102	11.3
	$3,000–$3,499	51	5.6
	$3,500–$3,999	23	2.5
	$4,000–$4,999	20	2.2
	$5,000 or more	40	4.4
	Prefer not to say	56	6.2
Past year gambling	None	305	33.7
	At least once	601	66.3
Past year EGM use (days in past year)	None	744	82.1
	1–10	97	10.7
	11–20	27	3.0
	21–49	9	1.0
	50 +	29	3.2
Problem gambling severity index	No harm	600	66.2
	Low risk	90	9.9
	Moderate risk	91	10.0
	High risk	125	13.8

### Policy Support

Participants were presented with a series of statements describing existing and proposed policies aimed at minimizing EGM-related harm. The policies selected were based on key recommendations by the Australian Productivity Commission (2010). Many of these policies have been widely discussed in the academic literature (Ladouceur et al., 2012; Livingstone & Woolley, 2007; Yücel et al., 2017), have been considered by Australian Parliamentary Committees (House of Assembly Select Committee on The Gaming Control Amendment Bill, 2010; Joint Select Committee on Gambling Reform, 2011), and have featured prominently in Australian media (e.g. Morton, 2018; Willingham, 2015). Policy items included proposals to limit the availability of EGMs, running mass media campaigns about gambling harm, providing clear in-venue information about counselling services or average hourly losses, providing free access to counselling services, self-exclusion and pre-commitment programs, and a maximum limit on EGM bets of $1 AUD per spin. Participants were asked to indicate the extent to which they agreed or disagreed using a labelled 6-level ordered response scale, (“Strongly Disagree”, “Disagree”, “Slightly Disagree”, “Slightly Agree”, “Agree”, “Strongly Agree”).

### Perceived Regulatory Compliance & Responsibility for EGM Related Harm

In addition to the policy items described above we included three items based on language from the Australian/New Zealand Gaming Machine National Standard and Australian Consumer Law. Finally, participants were asked whether a series of various stakeholders “should be held responsible when negative or harmful consequences occur as a result of poker machine use?” These items provided the same ordered response scale described above.

#### Hypotheses

Our analyses related to the first study aim were guided by the following set of exploratory hypotheses:

- Participants in the Design and Brain groups will attribute less responsibility for gambling-related harm to the individual gambler, relative to the Control and Industry conditions.
- The Design condition will attribute greater responsibility to industry and government.
- Participants in the Design group will report greater support for government interventions targeted at industry behaviour, machine or casino design, or access to gambling products, relative to the Control condition
- Participants in the Industry condition will report less support for government interventions targeted at industry behaviour, machine or casino design, or access to gambling products, relative to the Control condition.
- Participants in the Brain condition will report greater support for publicly funded counselling programs, compared to the other conditions.
- Finally, we sought to investigate whether our Brain condition would reduce participant support for harm prevention policy.

### Analysis

Our primary analyses employed Bayesian cumulative ordered probit models (Bürkner & Vuorre, 2019) that are more appropriate for the ordinal measurement instruments used in this study than a linear regression or ANOVA (Liddell & Kruschke, 2018). Each model included a single term for experimental condition and no covariates. We adopted mild regularising Normal(0, 0.5) priors for the condition parameter. This prior introduces some very mild scepticism about unfeasibly large differences in latent means but does not contain specific domain knowledge relating to the research questions. Normal(0, 1) priors were set for the model thresholds. Latent standard deviations were also allowed to vary by condition.

Standardised effect sizes are reported for all group-level contrasts to account for both the direction and magnitude of any group differences. These were calculated using the difference between model estimated latent means and pooled latent standard deviations, analogous to Cohen’s *d* (Cohen, 1988). All model estimates are provided along with Bayesian 95% highest density posterior intervals (HDPI) to indicate modelling uncertainty.

All analyses were performed in *R* (R Core Team, 2021). Plots were composed using *ggplot2* (Wickham, 2016) and *cowplot* (Wilke, 2020). Cumulative ordinal regression was performed using *brms* (Bürkner, 2017) and remaining analyses were performed using *rethinking* (McElreath, 2020), each of which provides a convenient interface for Bayesian modelling in *Stan* (Carpenter et al., 2017).

### Participants

Data collection occurred between 1st October 2020 and 12th November 2020. The median completion time was 9.6 min. Following standard quality control measures employed by the Qualtrics Research Team, 906 survey responses were collected from participants located in the Australian states of New South Wales (433) and Victoria (473). Group sizes were comparable after randomisation and data cleaning; Control = 234, Brain = 228, Design = 224, Industry = 220. Participant demographics are displayed in Table 1.

## Results

### Comprehension Check

The majority (69.4%) of participants responded correctly to the first comprehension check, while 10.7% asked to view the article again, and 19.9% answered incorrectly. Most respondents (87.7%) answered the first or second manipulation check correctly, representing a high level of comprehension of the material presented. A logistic regression indicated that the estimated pass rate (*p*_pass_) did not differ by group; Brain, *p*_pass_ = .87, 95% HDPI = [.82, .91]; Design, *p*_pass_ = .88, [.83, .92]; Industry *p*_pass_ = .88, [.82, .92]. Mean time spent reading each article was comparable across conditions after accounting for extreme outliers.

### Responsibility for EGM Related Harm

Total agreement (i.e., the proportion of participants who selected either Strongly Agree, Agree *or* Slightly Agree) for each responsibility item across the conditions is displayed in Table 2. Participants attributed greater responsibility to individuals, machine designers, government, and gambling venues, relative to social networks (i.e. friends and family), venue staff, and Australian society or culture in general.

**Table 2:** Observed Proportion Total Agreement and Model 95% HDPI for Responsibility Items by Group

Item	Control		Brain		Design		Industry	
	Obs.	HDPI	Obs.	HDPI	Obs.	HDPI	Obs.	HDPI
Individual	.89	[.86, .93]	.91	[.87, .94]	.93	[.90, .96]	.91	[.87, .94]
Social Network	.32	[.26, .37]	.33	[.27, .38]	.32	[.27, .38]	.33	[.30, .40]
Designers	.77	[.72, .82]	.77	[.73, .82]	.88	[.83, .90]	.73	[.68, .78]
Venue Owners	.78	[.75, .84]	.84	[.78, .87]	.88	[.84, .91]	.78	[.73, .83]
Venue Staff	.42	[.37, .47]	.37	[.33, .44]	.42	[.37, .48]	.37	[.31, .42]
Government	.78	[.74, .83]	.84	[.77, .86]	.89	[.83, .91]	.73	[.70, .80]
Aus Society	.62	[.56, .66]	.58	[.53, .64]	.59	[.55, .66]	.56	[.48, .60]

Effect size estimates for the difference between the latent means of each intervention condition and the Control were negligible or very small and all 95% HDPIs included both positive and negative values, *d*_Brain_ = 0.00, [− 0.19, 0.19], *d*_Design_ = 0.09, [− 0.10, 0.29], *d*_Industry_ = 0.08, [− 0.11, 0.27]. We also observed approximately equal endorsement across all contrasts involving the social network item, and between the Control condition and both the Design and Brain conditions, for the Australian culture item. Posterior medians for each of these effect sizes were within ± 0.05 of zero.

Participants in the Design group responded with a higher level of agreement that EGM designers (*d* = 0.27, 95% HDPI = [0.08, 0.46]), gambling venue owners (*d* = 0.27, [0.08, 0.46]) and government (*d* = 0.27, [0.07, 0.46]), should be held responsible for EGM-related harm, relative to the Control group and both the Industry and Brain conditions (all effect size estimates > 0.2). This greater endorsement of responsibility did not appear to spill over to venue employees (*d*_Design_ = − 0.2, [− 0.20, 0.17]). Differences in this instance were centred near zero, most consistent with a negligible or null effect, though the interval also included very small effect sizes.

We observed a slight reduction in the attribution of responsibility toward venue staff in the Brain condition, *d* = − 0.11, [− 0.30, 0.07], and Industry condition, *d* = − 0.18, [− 0.37, 0.00], although in both instances the HDPI included zero and near null values. Contrasts between the Brain and Control conditions for the remaining responsibility items, including those related to industry and government, were consistent with a negligible or very small effect of this intervention (*d* ≤ 0.05).

Model estimated effect sizes for the Industry intervention relative to the Control group suggested a slight reduction in the attribution of responsibility to Australian culture or society in general (*d* = − 0.15, [− 0.34, 0.03]), and, to a lesser extent, government (*d* = − 0.10, [− 0.29, 0.10]) and machine designers (*d* = − 0.11, [− 0.30, 0.07]), though HDPIs included zero and a range small effect sizes either side of zero. Finally, there was little to no effect for the venue owners’ item, (*d* = − 0.04, [− 0.22, 0.16]).

### Consistency of EGM Design with Regulatory Language

Response scales to these items included an “I Don’t Know” option, in addition to the 6 response levels used for the items above. The proportion of each group submitting an “I Don’t Know” response was less than 8% across each item and condition. See the supplementary materials for a more detailed treatment of these responses.

**Fig. 1:**
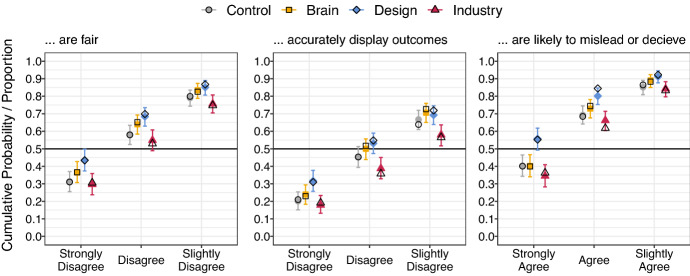
Observed cumulative response proportions and posterior median for cumulative probabilities for regulatory language items by group. These plots display *cumulative* proportions. This means that if the proportion who chose “Strongly Disagree” is displayed on the left, the following point on the x-axis indicates the proportion who selected either “Strongly Disagree” *or* “Disagree”, followed by any level of disagreement. Error bars indicate 95% highest density posterior intervals. Filled shapes indicate posterior medians. Unfilled shapes indicate observed cumulative proportions in data. Note that plots have been truncated to display the only first 3 response levels, and that the order of these response levels is reversed for the final item

There was broad agreement across all experimental groups (> 80%) that poker machines are likely to mislead or deceive consumers, see Fig. 1. Agreement with this item was greater in the Design condition, relative to the Control (*d* = .39 [0.18, 0.60]), Brain (*d* = 0.38 [0.16, 0.60]) and Industry conditions (*d* = 0.52 [0.29, 0.74]). There was little difference between the Brain and Control groups (*d* = 0.03 [− 0.17, 0.24]), and a very mild reduction to negligible difference in the Industry condition, relative to the Control (*d* = − 0.12 [− 0.34, 0.08]). Over half (observed = 55.3%, model estimate *p*_StronglyAgree_ = .557 [.493, 0.617]) of the participants in the Design group selected "Strongly Agree" on this item, and it remained the choice with the highest observed proportion across all groups, (Control = 40.1%, *p*_StronglyAgree_ = .402 [.343, .466]; Brain = 39.9%, *p*_StronglyAgree_ = .402 [.341, .465]; Industry = 36.3%, *p*_StronglyAgree_ = .344 [.281, .408]).

Conversely our sample tended to disagree that poker machines are fair (all groups > 70%). Strongly Disagree, was the most popular choice across all conditions, Control = 31.1%, *p*_StronglyDisagree_ = .313 [.256, .370]; Brain = 36.6%, *p*_StronglyDisagree_ = .365 [.308, .426]; Design = 43.4%, *p*_StronglyDisagree_ = .435, [.374, .499]; Industry = 30.6%, *p*_StronglyDisagree_ = .298 [.236, .355]. This tendency towards greater disagreement was more pronounced in the Design group, relative to both the Control group (*d* = − 0.30 [− 0.50, − 0.10]), and the Industry group (*d* = − 0.36 [− 0.56, − 0.15]). Participants in the Brain group also tended to disagree more, relative to the Industry (*d* = − 0.22 [− 0.43, − 0.02]), and Control conditions, (*d* = − 0.15 [− 0.34, 0.05]), and tended to agree more relative to the Design group (*d* = 0.16 [0.36, − 0.05]), though effect sizes were very small and HDPIs for the latter two estimates included both positive and negative values. Finally, differences between the Control and Industry group were small or negligible (*d* = 0.07 [− 0.13, 0.27]).

There was also majority disagreement across all groups with the statement “poker machines accurately display outcomes”. Relative to participants in the Industry condition, those in the Design and Brain conditions, indicated more disagreement, on average, *d*_IndustryDesign_ = − 0.35 [− 0.55, − 0.14], *d*_IndustryBrain_ = − 0.30 [− 0.50, − 0.09]. We also observed small differences relative to the Control condition; though all HDPIs included zero, and near zero values, *d*_Industry_ = 0.19 [− 0.01, 0.40]; *d*_Design_ = − 0.19 [− 0.39, 0.00]; *d*_Brain_ = − 0.12 [− 0.31, 0.08].

### Policy Support

#### Proposals to Limit the Availability of EGMs

**Fig. 2:**
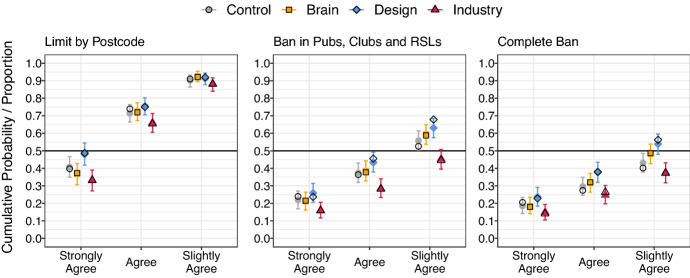
Observed cumulative response proportions, posterior median for cumulative probabilities and 95% HDPI for various proposals to limit EGM accessibility. As before these plots display *cumulative* proportions or probabilities (see Fig. 1 caption). Error bars indicate 95% highest density posterior intervals. Filled shapes indicate posterior medians. Unfilled shapes indicate observed cumulative proportions in data

Total agreement with each of the proposals to limit the availability of EGMs is displayed in Fig. 2. There was strong support across all conditions to limit the density of EGMs by postcode. There was slight majority support for the ban of EGMs in pubs and Industry in the Control group (the lower bound of the HDPI was at .504), and a majority for Brain, and Design Group, but not the Industry condition. Finally for a ban in all venues, including casinos, total agreement for the Design group was just above 0.5 (and the HDPI contained values below .5). For this last item, total agreement among the Industry and Control fell reliably below .5, and the Brain group was centred close to .5.

Posterior estimates of standardised effect sizes for all group contrasts are displayed in Table 3. Participants in the Industry condition displayed a greater tendency to disagree with proposals to limit access to EGMs, relative to all other conditions. Contrasts between the Design and Industry conditions were all reliably above 0.1, consistent with an increased tendency to agree with limiting access in the Design group relative to the Industry group. HDPIs also excluded zero for the proposal to limit access to EGMs in pubs and Industry, relative to the Control condition, and for both “pubs and clubs” and in all settings relative to the Brain condition.

**Table 3:** Posterior median and 95% HDPI for the effect size of each group contrast for EGM access items

Contrast	Postcode		Pubs and Clubs		Everywhere	
	50 %	HDPI	50 %	HDPI	50 %	HDPI
Brain − Control	− 0.06	[− 0.26, 0.14]	0.07	[− 0.12, 0.26]	0.15	[− 0.04, 0.33]
Design − Control	0.18	[− 0.03, 0.39]	0.17	[− 0.01, 0.37]	0.27	[0.10, 0.47]
Industry − Control	− 0.18	[− 0.38, 0.01]	− 0.27	[− 0.45, − 0.07]	− 0.14	[− 0.33, 0.04]
Brain − Industry	0.14	[− 0.07, 0.34]	0.35	[0.16, 0.55]	0.30	[0.11, 0.50]
Design − Industry	0.35	[0.14, 0.56]	0.44	[0.25, 0.64]	0.43	[0.23, 0.63]
Design − Brain	0.24	[0.02, 0.45]	0.11	[− 0.08, 0.30]	0.14	[− 0.05, 0.33]

Conversely, participants in the Design condition displayed a greater tendency to agree with proposals to limit access to EGMs relative to all other conditions, though contrasts with the Control and Brain conditions were typically small and HDPIs included zero or near zero values. The only exception was the proposal of a total ban on EGMs in all venues, where we observed a small effect size reliably above zero for the Design/Control contrast. Contrasts between the Brain and Control conditions were centred around small to negligible effect sizes, and all intervals included zero.

#### Pre-Commitment, Self-Exclusion and Other Policy Proposals

**Fig. 3:**
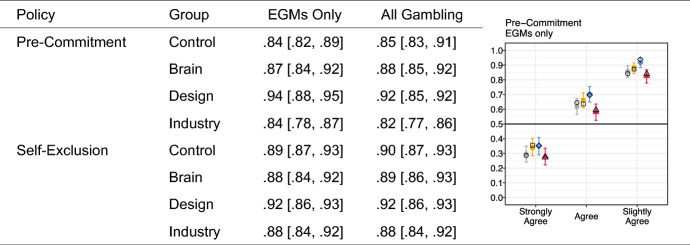
*Left* Table displays the observed proportion of total agreement alongside model 95% HDPI for self-exclusion and mandatory pre-commitment items. *Right* Plot displays observed cumulative response proportions, posterior median for cumulative probabilities and 95% HDPI for EGM pre-commitment. General response patterns across each of the self-exclusion and pre-commitment items were broadly similar, though the item shown here displayed the greatest between group variation. All plot aesthetics are mapped as in previous figures

There was widespread support for both pre-commitment and self-exclusion policies, see Fig. 3. Posterior estimates for median total agreement were above 80% for all groups, and total support was nearly identical, regardless of whether the policies was applied to EGMs only, or to all gambling products (including EGMs and online operators).

We observed no substantial difference between any condition for the self-exclusion items, posterior estimates for the effect size were either very small or negligible, and all HDPIs included a range of values either side of zero (see. Table 4). Likewise, differences for the pre-commitment items were small, and in most cases uncertainty intervals also included zero or near zero values. We observed a small difference between the Design and Industry condition for both settings, and a small difference between the Design and Control conditions for the application of pre-commitment to EGM venues. There was also a small difference between the Brain and Industry conditions for the pre-commitment proposals, although this HDPI included zero.

**Table 4:** Posterior effect size estimates for group contrasts for pre-commitment and self-exclusion items

Policy Proposal	Contrast	EGMs Only		All gambling	
		50 %	HDPI	50 %	HDPI
Pre-commitment	Brain − Control	0.12	[− 0.07, 0.31]	0.09	[− 0.11, 0.28]
	Design − Control	0.20	[0.00, 0.40]	0.13	[− 0.06, 0.33]
	Industry − Control	− 0.08	[− 0.27, 0.11]	− 0.08	[− 0.28, 0.11]
	Brain − Industry	0.19	[− 0.01, 0.38]	0.17	[− 0.03, 0.37]
	Design − Industry	0.27	[0.07, 0.48]	0.21	[0.01, 0.41]
	Design − Brain	0.07	[− 0.12, 0.27]	0.05	[− 0.16, 0.24]
Self-Exclusion	Brain − Control	0.01	[− 0.18, 0.21]	0.01	[− 0.18, 0.21]
	Design − Control	0.03	[− 0.16, 0.23]	0.02	[− 0.17, 0.22]
	Industry − Control	− 0.09	[− 0.29, 0.10]	− 0.08	[− 0.27, 0.12]
	Brain − Industry	0.10	[− 0.10, 0.30]	0.09	[− 0.11, 0.29]
	Design − Industry	0.12	[− 0.08, 0.32]	0.10	[− 0.11, 0.30]
	Design − Brain	0.02	[− 0.18, 0.22]	0.01	[− 0.18, 0.22]

Total agreement with the remaining policy proposals ($1 AUD maximum bets, free clinical treatment for gambling harm, mass media campaigns, and compulsory in-venue display of helpline contact information, expected hourly losses, and onscreen pop-up messages) was very high across all experimental groups, see supplementary materials.

## Discussion

### Strong Overall Support for Harm Minimisation Policy

In this study we sought to characterise the degree of community support for a series of prominent existing and proposed policies that aim to minimise gambling harm in a representative sample of Australian adults, living in New South Wales and Victoria. Overall, we observed a high level of total support for all policy proposals in this sample, the only exception being proposals to ban EGM gambling in bars and clubs, or in all settings including casinos. Proposals to limit access to EGMs also provoked more varied responding relative to other items. If we take the control group as an indicator of community attitudes independent of an intervention, there was bare majority support for a ban in clubs and pubs, but a bare majority against a complete ban that included casinos. Our results suggest a clear consensus in favour of all other policy interventions, including some more restrictive proposals such as $1 maximum bets on EGMs or mandatory pre-commitment schemes. This suggests a reasonable appetite in the community surveyed for policies intended to mitigate gambling harm, across all experimental conditions.

### Who Should be Held Responsible for EGM Related Harm?

Most participants agreed that an individual should be held responsible when their EGM gambling results in harmful consequences. Notably, a substantial majority of participants in all groups also agreed that state governments, EGM manufacturers and gambling venue operators should be held responsible for these harms. Consistent with our predictions, the Design condition responded with more agreement that EGM designers, venue owners, and state governments should be held responsible relative to all other conditions. Given that the role of government was not explicitly discussed in Design article, increased support on this item might suggest a perceived link between industry practice, and a role for government in mitigating or responding to any harm that occurs as a consequence of these strategies.

We found no support for our hypothesis that the Brain, or Design intervention would reduce agreement with statements suggesting that individuals be held responsible for their gambling-related harm. Given that we observed a clear effect of the Design intervention on the agreement that government and industry be held responsible, this may suggest that increased agreement with the responsibility of government or industry does not redistribute responsibility away from the individual, as though it were a limited resource. Aggregate responses also demonstrate that many individuals agreed that responsibility rested with multiple stakeholders. A caveat here is that our survey text specifically clarified that participants were “free to consider more than one actor responsible” for gambling related harm.

### EGMs are Perceived as Being Misleading, Deceptive, and Unfair

The Design group reported more agreement with the statement that poker machines are likely to mislead or deceive consumers, and more disagreement that poker machines are fair. This suggests that learning about EGM design features, such as LDWs, leads individuals to view EGM design as being misleading and deceptive, or unfair. We would also highlight that across all groups, responses to the items related to regulatory language suggest that EGMs are perceived by the community in a manner that is inconsistent with regulatory guidelines and consumer protection law in Australia. For the avoidance of doubt, these findings do not suggest or infer that EGMs or EGM design is misleading or deceptive within the meaning of the Australian Consumer Law. Rather, these findings suggest that current legal and regulatory oversight may be out of step with community attitudes or expectations. The observation that providing information about LDWs was associated with increased agreement that government should be held responsible for EGM related harm, might also suggest that the community sees government as responsible for intervening where harm might occur due to LDWs specifically. These findings may also hold some relevance for jurisdictions with comparable legislation or regulation, such as Canada where the Competition Act (R.S.C., c. C-34, s.52, 1985) contains provisions relating to “false or misleading” representations.

### How do Different Narratives of Gambling-Related Harm Influence Policy Support?

We hypothesised that the Design intervention would increase support for key policy items, while the Industry intervention would reduce support, relative to the Control. Most effect size estimates for the contrasts related to these hypotheses were small, and many HDPIs included zero, or values very close to zero. In these instances, results are also consistent with an intervention having little to no effect. For example, while all posterior effect size estimates for the Design/Control contrasts on policy proposal items were consistent with the direction of our predictions, only four of 13 HDPIs excluded negative values in (a ban on EGMs in all settings, introduction of mandatory pre-commitment for EGMs, free clinical treatment for gambling-related harm funded by gambling taxes, and mass media campaigns). There also appeared to be little to no influence of this intervention on support for self-exclusion policies. The only clear contrast between the Control and Industry condition was reduced support for the proposal to ban EGMs in pubs and clubs, though point estimates for the other proposals to limit access to EGMs were consistent with the direction of our predictions.

Contrasts observed for each policy item between the Design and Industry condition were typically larger than those considered above. Seven of the 13 HDPIs for these contrasts reliably excluded contrary values, and all point estimates were consistent with the direction of our predictions for differences between the Design and Industry conditions. These results do not warrant a definitive statement about whether our broad hypotheses related to policy support were substantiated. But they do provide tentative support for the predicted influence of the Design condition, and for a limited reduction in support for proposals to limit access to EGMs in the Industry condition.

We observed no clear evidence to support our prediction that participants who read our Brain intervention would be more likely to support counselling programs funded by gambling taxes, compared to the other conditions. The Design condition was the only group for which support on this item was reliably larger than the Control condition, although the effect size estimate was in the hypothesised direction for the Brain condition. We also sought to explore whether a description of the neuroscience of gambling addiction would decrease support for policy interventions targeted at the gambling environment or shift attributions of responsibility for gambling harm away from gambling products or the gambling industry. We found no evidence to suggest that this was the case. Broadly, model estimates for contrasts between the Brain and Control were most consistent with either a near null difference, and in some cases, a slight but uncertain *increase* in support for each proposal, providing tentative evidence that this intervention did not reliably or substantially *weaken* public support for gambling harm minimisation policy.

While this is the first study to have considered how various accounts of gambling related harm might influence support for public health policies, our findings are consistent with several studies that have investigated the link between causal attributions for over-weight and obesity with policy support. A number of cross-sectional studies have reported that endorsement of the view that the food environment contributes to obesity, positively predicts support for obesity prevention policy (Barry et al., 2009; Hilbert et al., 2007). Some studies have also reported that biomedical accounts of obesity are associated with increased support for policy intervention (Schulte et al., 2016). However, when compared with explanations endorsing the food environment, explanations that highlight biological influences on obesity are often reported to be associated with smaller effect sizes and support for a narrower range of policies (Barry et al., 2009; Beeken & Wardle, 2013). Pearl and Lebowitz (2014) reported an experimental study in which a brief explanation of environmental causes for obesity lead to stronger endorsement of obesity prevention policies as compared to a biological framing of obesity and a no intervention control condition. Although the current results relating to policy support do not support a conclusive statement on group differences, the general pattern of point estimates we observed is consistent with the obesity research summarised here. Whereby, relative to the Control, the Design condition responded with slightly more support, and the Brain condition typically fell somewhere between the Control and the Design groups.

### The Role of Neuroscience in Gambling Public Health and Clinical Treatment

There is substantial scope to align neuroscience research with a public health framing of gambling-related harm (Clark & Goudriaan, 2018; Myles et al., 2018; Yücel et al., 2017). A public health frame requires an account of the health-related impacts of gambling, including but not limited to the potential addictive influence of gambling products. Bio-behavioural research is well positioned to evaluate claims that product design or retailer practice can contribute to addictive behaviour. Further, an explanation of the biology underlying a condition is likely to be an essential component of clinical advice to patients and families. These narratives provide a means to highlight some of the diverse pressures on individuals experiencing difficulties. Given that this information may be necessary in some settings, the concern ought not be the deployment of neuroscience or clinical research per se, but rather with a narrow framing that obscures other important aetiologies of gambling related harm.

The present results suggest that the information about the neuroscience of gambling harm or addiction did not substantially undermine support for gambling-related public health interventions. The Brain intervention presented a simple account of the neuroscience of addiction. It did not situate this evidence within a wider social context, other than stating that these potentially addictive products are a source of substantial private profit. Nor did it explicitly make a case against any policy proposal. It remains feasible that neuroscience evidence may lend empirical credibility to an account of harmful influence for EGM design features, such as that offered in our Design condition, or to a wider public health framing of gambling-related harm (Myles et al., 2018). Likewise, it remains feasible that a biomedical frame deliberately deployed to obfuscate the wider antecedents of gambling-related harm, in favour of a narrow focus on severe cases of gambling disorder, may be an effective way to argue that policy changes that target features of the gambling environment are misguided, as argued in the Industry intervention (Yücel et al., 2017). The current results do not allow for a conclusive statement on such concerns, which remain a question for further research.

### Limitations and Future Directions

Most of the effect size estimates reported in this study were relatively small, and in many cases, we could not reliably exclude negligible or contradictory effects at current statistical power. We chose to employ mock news articles as they have a clear connection to the way information about social issues is typically consumed and considered, but this approach is unlikely to provoke large changes in individual attitudes. The influence of a single news article is likely small relative to volume of the information accrued over an individual’s lifetime, or a sustained and targeted media campaign. It is conceivable that the small effects observed in this study could accrue over a sustained campaign, or alongside other small but consistent effects (Funder & Ozer, 2019; Götz et al., 2022). Such campaigns may further amplify effects by starting wider conversations in the community. Additionally, media reporting and wider community conversations on a select issue will typically intensify when lawmakers are considering gambling reform. This may mean that the effects observed in the current study represents only a fraction of a typical “dose”. Alternatively, the effects observed in the present study may be quickly washed out amongst the volume of information consumed by the average Australian adult. A related consideration is that coverage of gambling related issues is not uncommon in Australian media (Boyce, 2019; Evershed et al., 2017; Manning, 2015; Puddy, 2022). This may mean that participants in each of our experimental conditions had already been frequently exposed to some version of the information presented within each of our interventions, limiting the effectiveness of the experimental design.

Further research investigating these issues should consider the need to further increase statistical power to better rule out or corroborate small effects. The effect size estimates reported in the current study will enable power analyses or simulation studies to calculate necessary sample size. Future studies could also consider alternative methods—such as videos of vignettes—that might be more efficacious or engaging than a news article. Future studies could also increase statistical power through repeated measures of key outcomes. For example, media-based interventions such as those used in the present study, could consider pre-post testing while vignette studies offer another attractive option for repeated measurement (Baguley et al., 2021; Wallander, 2009).

Any conclusions drawn about the influence of the articles used in this study, may not generalise to thematically comparable narratives deployed in different ways. For instance, the impact of any information may vary in its influence depending on, inter alia, the medium used, the perceived credibility of the source, or the specific examples or arguments deployed in support a perspective. An important consideration here is that lobbying campaigns are typically targeted at specific policy proposals. In the case of our Industry intervention, we did not include references to specific policies, which might have mitigated the potential influence on specific policies. We would also note that the Industry intervention was presented as a media-release on the website of an interested party, rather than a trusted news source.

Finally, despite our efforts to recruit a broadly representative sample, these findings may not adequately generalise beyond the sample recruited to this study, that is, people from the Australian states of New South Wales and Victoria who participate in online research panels. While our sampling was stratified on coarse demographics, we have not applied weighted adjustments on our estimates. As a result, our observations may overestimate or underestimate the scale of community support for these issues. We would note, however, that any sampling bias would need to be very substantial to offset the large proportions of total agreement observed across all groups for many of the items in the study.

### Supplementary Information

Below is the link to the electronic supplementary material.

Supplementary file1 (DOCX 228 KB)

## Data Availability

De-identified raw data, processed data, data analysis scripts, and materials used in this experiment have been made available on the Open Science Framework and GitHub. HTML/CSS scripts used to reproduce the appearance of the website of a major newspaper have not been included following concerns raised by our university legal department about to how closely they resembled the original publication. There were additional concerns raised about the name chosen for the lobby group in the Industry intervention, so this has also been removed from source materials. However the essential text and primary images used in these articles has been included, as have all questionnaire items. OSF Project: https://osf.io/b8a9f/.

## Abbreviations

DSM 5: Diagnostic and statistical manual of mental disorders, fifth edition (American Psychiatric Association)
EGM: Electronic gambling machine
GD: Gambling disorder
HDPI: Highest density posterior interval
LDW: Losses disguised as wins
OSF: Open science framework

## Code Availability

OSF Project: https://osf.io/b8a9f/.
