# Supplementary material for: Three Contrasting Accounts of Electronic Gambling Machine Related Harm: Impacts on Community Views Towards Gambling Policy and Responsibility
Source: J Gambl Stud. 2023 Apr 28;40(1):29–49. doi: 10.1007/s10899-023-10206-1 (PMC10904407; doi:10.1007/s10899-023-10206-1)
Supplement: Supplementary file 1 — Supplementary file1 (DOCX 228 KB) [file 10899_2023_10206_MOESM1_ESM.docx]

# Supplementary Materials

Three Contrasting Accounts of Electronic Gambling Machine Related Harm: Impacts on Community Views Towards Gambling Policy and Responsibility

Table of Contents

[Supplementary Materials 1](#_Toc105159757)

[Survey Items 1](#_Toc105159758)

[Policy Proposals 1](#_Toc105159759)

[Perceived Responsibility for EGM Related Harm 2](#_Toc105159760)

[Supplementary Analyses 4](#_Toc105159761)

[I Don’t Know Responses to Regulatory Language Items 4](#_Toc105159762)

[Remaining Policy Proposals 5](#_Toc105159763)

## Survey Items

### Policy Proposals

**Proposals to Limit the Availability of EGMs:**

- "State governments should impose a limitation on the number of poker machines available in any one postcode, to prevent the clustering of machines in disadvantaged areas."
- "Pokies gambling should be banned in pubs, clubs and RSLs."
- "Pokies gambling should be banned in all venues including casinos."

**Public perception of regulatory compliance:** These statements are based directly on language that appears in national regulatory guidelines for poker machine design (Australian/New Zealand Gaming Machine National Standard, 2016, p. 12) and Australian consumer protection law (Australian Consumer Laws (ACL), 2010). Response options for these items also included “I Don’t Know”. These statements were:

- "Poker machines are fair"
- "Poker machines accurately display gambling outcomes"
- "Poker machines are likely to mislead or deceive consumers"

**Media campaigns:**

- “Governments should run mass media campaigns (advertisements on billboards, television or radio) funded by taxes on gambling revenue that provide information about gambling harm?”

**In-venue information:** These items proposed that gambling venues, as a condition of their license, should be required to:

- “… display prominent warnings and contact information for gambling counselling services inside gambling venues in clearly visible locations”
- “… prominently display accurate information about the average hourly losses of poker machines”
- “… display pop-up messages designed to prevent harmful gambling on the poker machine screen when an individual has been using a machine for an extended period of time”

**Access to counselling:**

- **“**Australians should have access to counselling and treatment for gambling addiction at no cost, funded by taxes on gambling revenue.”

For both pre-commitment and self-exclusion questions we first displayed a short explanation of each program.

**Mandatory Pre-commitment:** A mandatory pre-commitment program is a gambling harm reduction strategy that requires anyone who wishes to gamble to set a maximum daily or weekly limit on the amount they can spend gambling using a unique card or account. They are free to choose any amount as their limit. If they wish to change that limit, they would have to wait until a commitment period had ended before the new limit would come into effect. These programs are intended to help individuals maintain self-control over the amount that they feel they can afford to spend.

Under this program, a condition of the license required to operate a gambling venue (or service) is that the venue must refuse to serve anyone who has exceeded their own set limit. All venues would have to use the same central system, so a player could not move to another venue once their limit was reached.

The Australian Federal Government should introduce …

- A mandatory pre-commitment scheme:
  - … for all pokies gambling venues
  - … for pokies gambling venues *and* all other gambling venues (including online operators)?

**Self-exclusion:** A gambling self-exclusion program is a gambling harm reduction strategy in which an individual can voluntarily elect to restrict their access to gambling venues or online providers. It is then the responsibility of a gambling operator to refuse service to any patron who has registered for self-exclusion. This requires operators to request each individual's identification and check their name and date of birth against a central self-exclusion record.

The Australian Federal Government should introduce …

- A self-exclusion program:
  - … for all pokies gambling venues
  - … for pokies gambling venues *and* all other gambling venues (including online operators)?

**Maximum bets:** This item was included alongside an explanation that at-risk gamblers tend to gamble with larger amounts of money than recreational gamblers. We then asked participants whether they agreed or disagreed that “governments should limit the maximum bet on all poker machines to no more than $1 per spin?”

### Perceived Responsibility for EGM Related Harm

These items asked participants “to what extent do you agree or disagree that each of the following actors should be held responsible when negative or harmful consequences occur as a result of poker machine use?”.

The complete list of actors was as follows:

- The individual should be held responsible
- The individual’s immediate family or close friends should be held responsible
- The companies or individuals who design and sell poker machines to venues should be held responsible
- The companies or individuals who own and profit from casinos and pokies venues should be held responsible
- The individual employees who work in gambling venues, such as bar staff, floor managers, dealers or croupiers should be held responsible
- State governments who legalise, regulate and permit gambling should be held responsible
- Australian society or culture in general should be held responsible

## Supplementary Analyses

### I Don’t Know Responses to Regulatory Language Items

Response scales to these items also included an “I Don’t Know” option, in addition to the 6 response levels used for the items above. Prior to analysing cumulative agreement with each of these items, we first sought to establish whether there was a systematic tendency to respond with "I Don't Know" by group. This analysis employed three separate Bayesian logistic regression models, with index coded variables for each of the experimental groups. Index coding provides the benefit of being able to set the same Logistic(0, 1) prior across conditions, which corresponds to an approximately uniform prior on the probability scale.

The table below contains the output for this analysis. The p columns display the estimated probability of a response by group and item (i.e. the probability a participant selected "I Don't Know"), along with 95% HDPIs. The OR columns contain the odds ratio for responding relative to the Control condition for each item.

Table 1 - Model Summary for I Don't Know Responses

| Item | Group | p_IDK_ | OR |
| --- | --- | --- | --- |
| Fair | Control | .064 [.035, .096] |  |
|  | Brain | .018 [.003, .034] | 0.281 [0.039, 0.609] |
|  | Design | .022 [.005, .041] | 0.360 [0.065, 0.744] |
|  | Industry | .064 [.034, .096] | 1.077 [0.371, 1.946] |
| Display | Control | .124 [.083, .166] |  |
|  | Brain | .101 [.065, .141] | 0.818 [0.405, 1.325] |
|  | Design | .063 [.032, .094] | 0.489 [0.193, 0.830] |
|  | Industry | .123 [.080, .164] | 1.022 [0.506, 1.617] |
| Mislead | Control | .051 [.024, .079] |  |
|  | Brain | .022 [.005, .041] | 0.454 [0.078, 0.980] |
|  | Design | .022 [.005, .042] | 0.462 [0.072, 0.987] |
|  | Industry | .073 [.038, .107] | 1.590 [0.536, 2.918] |

This analysis suggested that individuals in the Design and Brain groups were less likely to respond "I Don't Know" to whether they thought that "Poker machines are fair", relative to the Control condition. Participants in the Design group were also less likely to respond "I Don't Know" to whether or not EGMs accurately displayed outcomes, relative to the Control condition, whereas the odds of responding following reading the Brain group intervention were closer to even on this item and the 95% HDPI included values either side of 1. The relative odds of "I Don't Know" responses to the item "Poker machines are likely to mislead or deceive consumers" were approximately 1:2 relative to the Control for both the Brain and Design group, although the upper bound of the HDPI included values close to one in each case. The Industry condition did not differ substantially from the Control condition in their tendency to express an opinion on any item. The odds were close to even for the Fair and Display item, whereas the odds of an "I Don't Know" response to the item: "Poker machines are likely to mislead or deceive consumers" were slightly higher relative to the Control condition, though the HDPI the included values either side of 1. These results provide some tentative evidence that our Design and Brain intervention encouraged participants who might otherwise felt unable to provide a response, to respond on these items.

### Remaining Policy Proposals

Overall, total agreement with each of the remaining policies proposals not discussed in the primary results section was very high across all experimental groups, see **Fig. 1**. Around 90% of our sample supported displaying in-venue information about gambling harm, counselling services or the average hourly loss rate associated with in venue EGMs, as well as EGM pop-up messages. Total support for a $1 maximum limit on EGM bets, access to free gambling counselling or treatment, and government mass media campaigns was also very high (>75%), see supplementary materials.​

**Fig. 1** Observed cumulative response proportions, posterior median for cumulative probabilities and 95% HDPI for remaining policy proposals. These plots display cumulative proportions or probabilities (see paper, Fig. 1 caption). Error bars indicate 95% highest density posterior intervals. Filled shapes indicate posterior medians. Unfilled shapes indicate observed cumulative proportions in data.

Contrasts between each experimental group and the control group are displayed in **Fig. 1**. Relative to the Control condition, respondents who read our Design intervention responded with greater support for the hypothecation of gambling taxes to fund free counselling or treatment for gambling-related harm, and mass media campaigns to raise awareness about gambling-related harm.

Effect size estimates for contrasts on the remaining policy items were either negligible or small and HDPIs included zero. We had hypothesised that the Brain condition would be most likely to endorse the proposal for free counselling or clinical treatment, relative to all other groups. Contrary to this expectation, support for this proposal was highest in the Design group, though the effect size estimate for the contrast between the Brain and Design condition was very small *d* = 0.07 [-0.13, 0.26]. Support for each of these measures in the Industry condition, was typically close to that of the Control condition, with the possible exception of displaying expected hourly losses on or near EGMs in venues, for which this group responded with slightly less support overall. Although the HDPI included zero in that instance, we did observe a more substantial effect size, relative to the Design condition d = 0.36 [0.15, 0.57]. There was also slightly more support for $1 maximum bets in both the Brain and Design groups, relative to the control group, though HDPIs for each of these contrasts included zero.

Table 2. Posterior Effect Size Estimates for Contrasts with the Control Condition for Remaining Items

| Item | Brain - Control | Design - Control | Industry - Control |
| --- | --- | --- | --- |
| $1 AUD Maximum Bets | 0.19 [-0.01, 0.39] | 0.18 [-0.02, 0.38] | 0.04 [-0.16, 0.24] |
| Free Treatment | 0.15 [-0.04, 0.34] | 0.22 [0.03, 0.41] | 0.10 [-0.10, 0.29] |
| Media Campaigns | 0.14 [-0.05, 0.33] | 0.20 [0.00, 0.39] | -0.02 [-0.21, 0.17] |
| Helpline and Warnings | 0.10 [-0.11, 0.29] | 0.15 [-0.05, 0.35] | 0.03 [-0.16, 0.24] |
| Expected Hourly Losses | -0.01 [-0.21, 0.19] | 0.14 [-0.06, 0.35] | -0.19 [-0.38, 0.01] |
| Onscreen Pop-Up Messages | 0.05 [-0.16, 0.25] | 0.11 [-0.10, 0.32] | -0.04 [-0.24, 0.16] |

Table 3. Posterior Effect Size Estimates for Contrasts Between Experimental Conditions for Remaining Items

| Item | Brain - Industry | Design - Industry | Design - Brain |
| --- | --- | --- | --- |
| $1 AUD Maximum Bets | 0.13 [-0.07, 0.34] | 0.13 [-0.07, 0.33] | -0.01 [-0.20, 0.21] |
| Free Treatment | 0.05 [-0.15, 0.24] | 0.12 [-0.07, 0.32] | 0.07 [-0.13, 0.26] |
| Media Campaigns | 0.16 [-0.04, 0.35] | 0.22 [0.02, 0.42] | 0.06 [-0.14, 0.25] |
| Helpline Number and Warnings | 0.06 [-0.15, 0.27] | 0.11 [-0.09, 0.33] | 0.05 [-0.16, 0.26] |
| Expected Hourly Losses | 0.20 [-0.01, 0.40] | 0.36 [0.15, 0.57] | 0.16 [-0.06, 0.37] |
| Onscreen Pop-Up Messages | 0.09 [-0.12, 0.30] | 0.16 [-0.05, 0.37] | 0.07 [-0.15, 0.28] |

​
